# Proliferative ductular reactions correlate with hepatic progenitor cell and predict recurrence in HCC patients after curative resection

**DOI:** 10.1186/2045-3701-4-50

**Published:** 2014-08-27

**Authors:** Fei Ye, Ying-Ying Jing, Shi-Wei Guo, Guo-Feng Yu, Qing-Min Fan, Fang-Fang Qu, Lu Gao, Yang Yang, Dong Wu, Yan Meng, Feng-Hai Yu, Li-Xin Wei

**Affiliations:** Tumor Immunology and Gene Therapy Center, Eastern Hepatobiliary Surgery Hospital, The Second Military Medical University, Shanghai, China; Department of Hepatic Surgery, Eastern Hepatobiliary Surgery Hospital, The Second Military Medical University, Shanghai, China; Department of Radiation Oncology, Eastern Hepatobiliary Surgery Hospital, the Second Military Medical University, Shanghai, China; Department of Gastroenterology, Eastern Hepatobiliary Surgery Hospital, the Second Military Medical University, Shanghai, China

**Keywords:** Proliferative index of ductular reaction, Hepatic progenitor cell, Recurrence, Hepatocellular carcinoma

## Abstract

**Background:**

Ductular reactions (DRs) are well documented in many acute and chronic liver disease.The DRs are thought to be the transit amplifying cells deriving from activation of the stem/progenitor cell compartments of the liver. The aim of this study was to examine the presence of proliferative index of DR (PI-DR) and HPC markers’ expression in HCCs after curative hepatectomy, as well as their relationship with clinicopathological features and prognosis.

**Results:**

Tissue microarray with peritumoral and intratumoral tissue samples of 120 HCCs after hepatectomy was analysed for peritumoral expression of proliferating cell nuclear antigen for PI-DR. Peritumoral and intratumoral expression status of HPC markers including EpCAM, OV6, CD133 and c-kit were also examined by immunohistochemistry. TMA analysis of HCCs revealed that peritumoral PI-DR strongly correlated with the degree of inflammation and fibrosis. The peritumoral PI-DR positively correlated with peritumoral HPC markers EpCAM, OV6, CD133 and c-kit expression. Moreover, there were highly significant correlations between peritumoral PI-DR and intratumoral HPC markers EpCAM, OV6, CD133 and c-kit expression. Further, multivariate analysis showed that peritumoral PI-DR was the independent prognostic factor for overall survival (HR; 3.316, *P* < 0.001), and peritumoral PI-DR had a better power to predict disease-free survival (HR; 2.618, *P* < 0.001).

**Conclusions:**

Peritumoral PI-DR, as a valid surrogate for peritumoral and intratumoral expression of HPC markers, could be served as a potential prognostic marker for recurrence and survival in HCC after hepatectomy.

## Introduction

Hepatocellular carcinoma (HCC) is one of the main concerns in global health care which is difficult to cure because of recurrence after resection [1, 2]. Most research has been focused on the discovery of specific markers useful for HCC diagnosis and prognostic prediction to provide scientific guidance to clinical management [3, 4]. Recently, a number of investigations have demonstrated that the involvement of a ductular reaction (DR), which is a reactive lesion at the portal tract interface comprising increased bile ductules with an accompanying complex of stromal and inflammatory cells, has been implicated in the pathogenesis of progressive fibrosis, regeneration and hepato-carcinogenesis in chronic liver disease [5, 6]. The main epithelial component of DR are reactive ductule cells (RDCs) with biliary/HPC phenotype arranged in an irregularly shaped structure residing along parenchymal-stromal boundaries. The RDCs are pathological, proliferative, biliary epithelial cell-like cells formed during repair process after liver injury, and are able to secrete various cytokines and chemokines, which interact with microenvironment [7]. In previous studies, we have reported that peritumoral DR in a necroinflammatory microenvironment was a poor prognostic factor for HCC after resection [8]. However, the role of the proliferation status of DR remains elusive in HCC.

Ductular reaction is known to be present in most chronic liver diseases, but it seems especially important in hepatic stem and progenitor cells in liver regeneration mechanisms underlying hepatic fibrosis, and hepatobiliary carcinogenesis. Although not well described, however, when there is either massive liver injury or chronic liver damage that compromise the proliferative capacity of hepatocytes, progenitor cells within the Canal of Hering start to proliferate, giving rise to what is known as ductular reaction [9, 10]. The hepatic progenitor cells (HPCs) have been identified in the human liver as bipotential cells capable of proliferation and differentiation into both hepatocellular and biliary cell lineages [11, 12]. HPCs are reportedly recognized as cells smaller than normal hepatocytes, and present as a single cell or strings of a few such cells in periportal or periseptal regions of regenerative nodules or hepatic lobules. HPCs express biliary markers (such as OV6 and EpCAM), and a subset of these cells expresses hematopoietic markers (such as the receptor for stem cell factor c-kit [KIT] and prominin-1 [PROM1]/CD133) [13–16]. In additon, HCCs expressing HPC markers are likely to have significantly more negative prognosis and a higher recurrence after surgical resection and liver transplantation [17, 18]. To the best of our knowledge, the significance of the proliferative DR (PI-DR) and the correlation between PI-DR and the expression of HPC markers in terms of HCC prognosis has not been studied in detail.

Therefore, we performed a clinicopathological study on 120 cases who had undergone hepatectomy for histologically proven HCC, using the tissue microarray containing paired peritumoral and intratumoral liver specimens, we quantified the proliferative index of DR (PI-DR) in the peritumoral tissue. In addition, immunohistochemical detection of peritumoral and intratumoral HPC markers were performed to investigate the relationship between PI-DR and HPCs in detail. Moreover, we further explore the role of PI-DR in the HCC prognostic prediction after curative resection and their clinical relevance.

## Results

### Clinico-pathological features

The clinical and histological data are summarized in Table 1. Among the 120 subjects with pathological specimens available for assessment, 108 (90.0%) were male. This table also details the distribution of subjects within each grade of inflammation and stage of fibrosis. At the time of the last follow-up, 111 patients had tumor recurrence and 76 patients had died, and all patients died with a record of tumor recurrence. Among patients undergoing curative resections (n =120) the 1-, 3-, and 5-year overall survival (OS) rates were 92.3%, 46.4%, and 32.6%, respectively, and the 1-, 3-, and 5-year disease-free survival (DFS) rates were 70.7%, 14.7%, and 9.6%, respectively.

**Table 1:** **Demographic and baseline characteristics of 120 patients with hepatocellular carcinoma**

Factors	Value	Percent
**Demographic characteristics**		
Age	50.1 ± 11.9	
Gender (male/female)	108/12	90.0/10.0
**Tumor characteristics**		
Maximum tumor diameter	6.9 ± 4.2	
Tumor encapsulation	33	27.5
Tumor number (multiple)*	15	12.5
Microvascular invasion	65	54.2
Major vascular invasion	6	5.0
Edmondson grade		
Low (I/II)	90	75.0
High (III/IV)	30	25.0
TNM stage		
I/II	92	76.7
III	28	23.3
**Histology of chronic liver disease**		
Necroinflammation grade (score)		
No (0)	0	0.0
Minimal (1–4)	6	5.0
Mild (5–8)	38	31.7
Moderate (9–12)	57	47.5
Marked (13–18)	19	15.8
Fibrosis stage		
0	4	3.3
1-4	67	55.8
5-6	49	40.9
**Laboratory values**		
AFP (ng/mL)	351.0 ± 414.1 (1.7-1000.0)	
CEA (ng/mL)	4.3 ± 7.1 (0.0-58.2)	
CA19-9 (U/mL)	15.8 ± 14.4 (0.0-120.8)	
ALT (U/L)	54.6 ± 37.2 (9.8-234.9)	
AST (IU/L)	39.5 ± 19.4 (2.0-108.3)	

*Abbreviations*: *AFP* alpha-fetoprotein; *CEA* carcinoembryonic antigen; *CA19-9* carbohydrate antigen 19–9; *ALT* alanine aminotransferase; *AST* aspartate aminotransferase; *TNM* tumor-node-metastasis.

*Multifocal tumors diagnosed by preoperative image finding.

### Peritumoral proliferative ductular reaction (PI-DR) is present in HCC and correlates with the degree of inflammation and fibrosis

**Figure 1:**
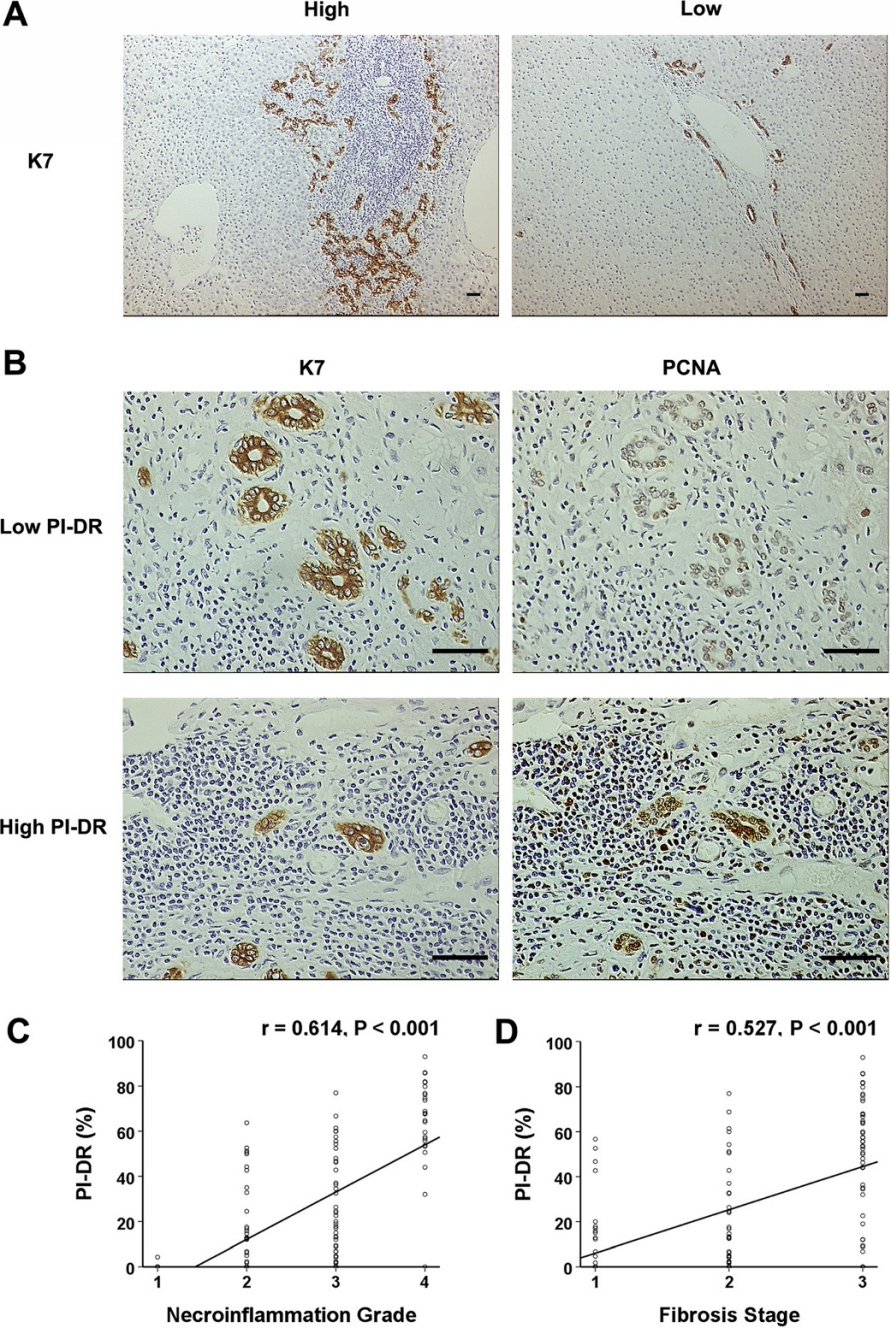
**PCNA labeling index of ductular reaction** **(PI-** **DR)** **positively correlated with the necroinflammation grade and fibrosis stage.** **(A)** The K7 immunostaining ductular reaction seen with examples in peritumoral tissue of hepatocellular carcinoma (sacle bar = 100 μm). Increased numbers of ductular reactions (≥50%) are noted at the edge of the portal tract containing a lymphoid aggregate (left), whereas continuous ductular reactions (<50%) are present along the edge of an enlarged portal tract (right). **(B)** Light micrograph of area displaying PCNA staining for DR represents a low level of proliferation activity (upper right) and a high level of proliferation activity (lower right), as corresponding with serious K7 staining of DR (sacle bar = 100 μm). **(C)** A split scattergram with fitting line shows peritumoral PI-DR correlates with necroinflammation grade in peritumoral tissue. **(D)** The peritumoral PI-DR is significantly correlated with fibrosis stage. Spearman correlation analysis provides correlation coefficient (r) and *P*-value.

As reported, the ductular reaction (DR) was positive uniformly and strongly for cytokeratin 7 staining [12]. In peritumoral K7 staining sections of the entire tissue microarray group, DR was frequently found in the fibrous septa and the enlarged periportal areas in HCC patients (Figure 1A). Further, In terms of PI-DR, the proliferating cell nuclear antigen (PCNA) was labeled for the proliferation status of DR (Figure 1B). 37 of 120 patients (30.8%) had PI-DR ≥50%.

Previous studies have shown that DRs correlate with the degree of inflammation and fibrosis in the course of many chronic human liver diseases [9, 10]. Thus, we evaluated the liver necroinflammation and fibrosis and their association with PI-DR. Consistent with prior studies, we observed PI-DR correlated with hepatic necroinflammation grade (r = 0.614, *P* < 0.001, Figure 1C). Correlations were also found between PI-DR and fibrosis stage (r = 0.527, *P* < 0.001, Figure 1D).

### Expression of hepatic progenitor cell markers in peritumoral tissue of HCC patients and clinicopathologic correlations:relationship with peritumoral PI-DR

**Figure 2:**
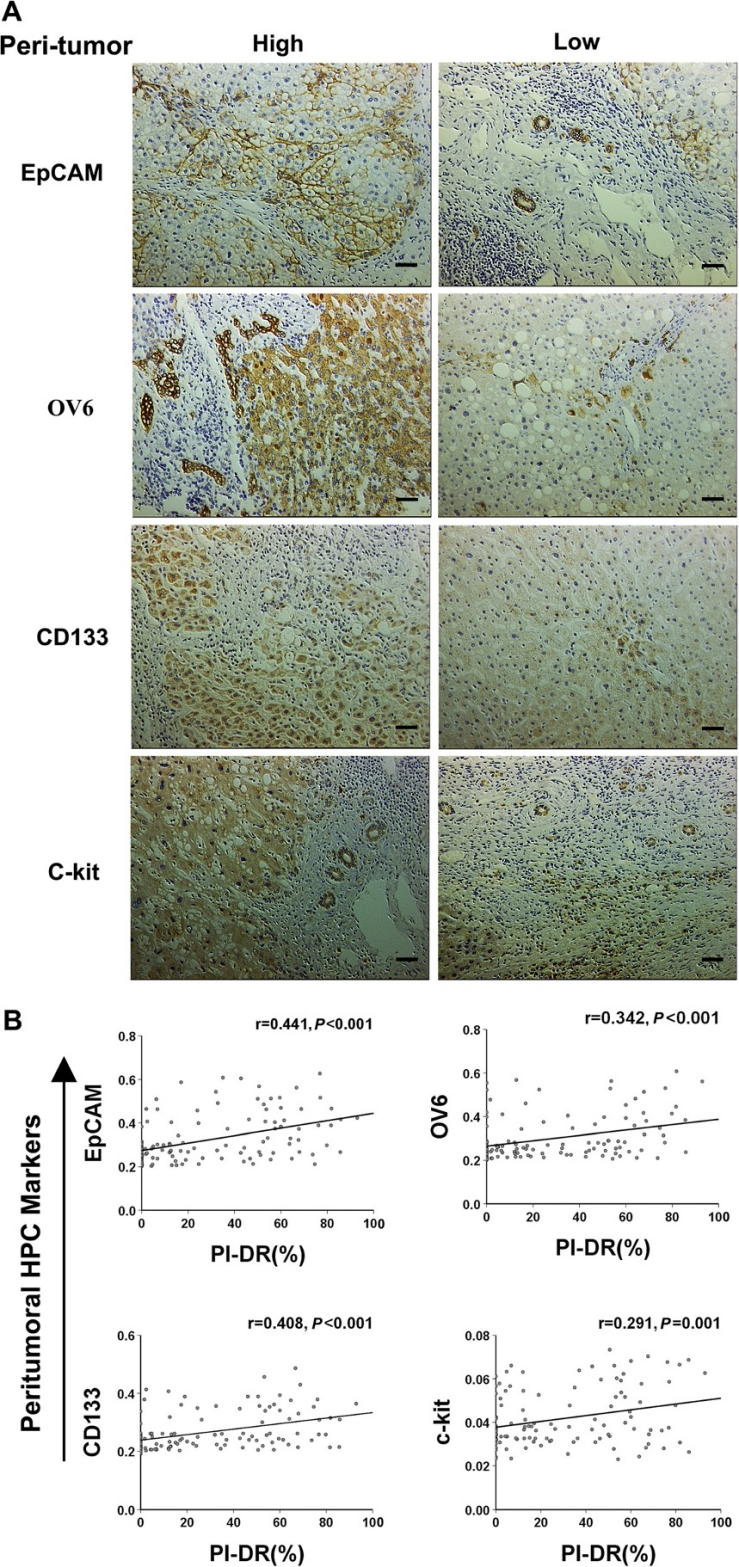
**The peritumoral PI**-**DR positively correlates with the peritumoral expression of HPC markers.** **(A)** Photographs of immunostaining of hepatic progenitor cell markers in peritumoral sections of tissue microarrays were taken for analyses (sacle bar = 100 μm). High and low density of EpCAM, OV6, CD133 and c-kit expression in peritumoral sections. **(B)** Scatterplot with fitting line shows peritumoral PI-DR positively correlates with peritumoral EpCAM, OV-6, CD133 and c-kit expression measured by IOD. Pearson correlation analysis provides correlation coefficient (r) and *P*-value.

Several markers, including EpCAM, OV6, CD133 and c-kit, which are expressed as markers for hepatic progenitor cells (HPCs), have been considered to be the origin of HCCs [19]. However, the link between PI-DR and expression of HPC markers has not been fully elucidated. We firstly conducted an immunohistochemical analysis of four HPC markers (EpCAM, OV6, CD133 and c-kit) in the peritumoral tissue sections of the entire group, the high EpCAM, OV6, CD133 and c-kit expression was seen in 40 of 120 (33.3%), 34 of 120 (28.3%), 33 of 120 (27.5%), and 39 of 120 (32.5%) cases, respectively (Table 2). EpCAM expression was seen in the membrane and cytoplasm of cholangiocytes of all branches of the biliary tree, including canals of Hering, ductules, and small and large bile ducts. The ductular OV-6-positive cells with a more transitional morphology, represented small numbers of individual oval-like cells adjacent to ducts in marginal regions. CD133-positive cells were mostly accumulated as patches with granular appearance in the cytoplasm of a few scattered hepatocytes. C-kit positive cells were diffused in the parenchyma in HCCs (Figure 2A).

**Table 2:** **Immunohistochemical stain results and clinicopathologic features in peritumoral tissue of 120 HCCs**

Clinicopathologic features	Peritumoral EpCAM density		***P***Value	Peritumral OV6 density		***P***Value	Peritumral CD133 density		***P***Value	Peritumral c-kit density		***P***Value
	High	Low		High	Low		High	Low		High	Low	
Frequency (%)	40 (33.3%)	80 (66.7%)		34 (28.3%)	86 (71.7%)		33 (27.5%)	87 (72.5%)		39 (32.5%)	81 (67.5%)	
Gender												
Male	35	73	0.747	30	78	0.946	30	78	1.000	37	71	0.363
Female	5	7		4	8		3	9		2	10	
Age												
≤50	21	42	1.000	16	47	0.453	19	44	0.493	20	43	0.853
>50	19	38		18	39		14	43		19	38	
AFP												
≤400	25	56	0..408	22	59	0.681	23	58	0.752	24	57	0.333
>400	15	24		12	27		10	29		15	24	
Cirrhosis												
Absence	20	51	0.149	19	52	0.645	17	54	0.294	17	54	0.016*
Presence	20	29		15	34		16	33		22	27	
Satellite lesion												
Absence	36	69	0.770	28	77	0.284	29	76	1.000	35	70	0.606
Presence	4	11		6	9		4	11		4	11	
Tumor size (cm)												
≤5cm	15	37	0.362	15	37	0.913	16	36	0.483	18	34	0.665
>5cm	25	43		19	49		17	51		21	47	
Tumor encapsulation												
Absence	26	61	0.193	26	61	0.540	23	64	0.672	27	60	0.578
Presence	14	19		8	25		10	23		12	21	
Microvascular invasion												
Absence	17	38	0.604	17	38	0.565	18	37	0.238	22	33	0.107
Presence	23	42		17	48		15	50		17	48	
Major vascular invasion												
Absence	39	75	0.657	33	81	0.853	29	85	0.083	38	76	0.687
Presence	1	5		1	5		4	2		1	5	
Edmondson grade												
Low (I/II)	27	63	0.180	21	69	0.035*	23	67	0.409	28	62	0.574
High (III/IV)	13	17		13	17		10	20		11	19	
TNM stage												
I/II	25	67	0.009*	23	69	0.142	16	76	<0.001*	29	63	0.678
III	15	13		11	17		17	11		10	18	

*p <0 .05 was considered statistically significant.

HCCs were grouped according to the expression status of peritumoral HPC markers, and clinicopathological parameters were further analyzed. As shown in Table 2, patients with a high peritumoral EpCAM and CD133 expression level were more prone to have high TNM stage, high Edmondson grade in patients with the high peritumoral OV6 expression, and high peritumoral c-kit expression patients demonstrated more cirrhosis.

Next, we further identify the correlation between PI-DR and the peritumoral HPC markers’ expression (IOD). PI-DR had a positive correlation with EpCAM (r = 0.441, *P* < 0.001), OV6 (r = 0.342, *P* < 0.001), CD133 (r = 0.408, *P* < 0.001) and c-kit expression (r = 0.291, *P* = 0.001) (Figure 2B) in peritumoral tissue of the entire group. These findings support the concept that proliferative DR in peritumoral environment may contribute to activation of progenitor cells and related carcinogenesis.

### Peritumoral PI-DR as a valid surrogate for intratumoral expression of HPC markers

**Figure 3:**
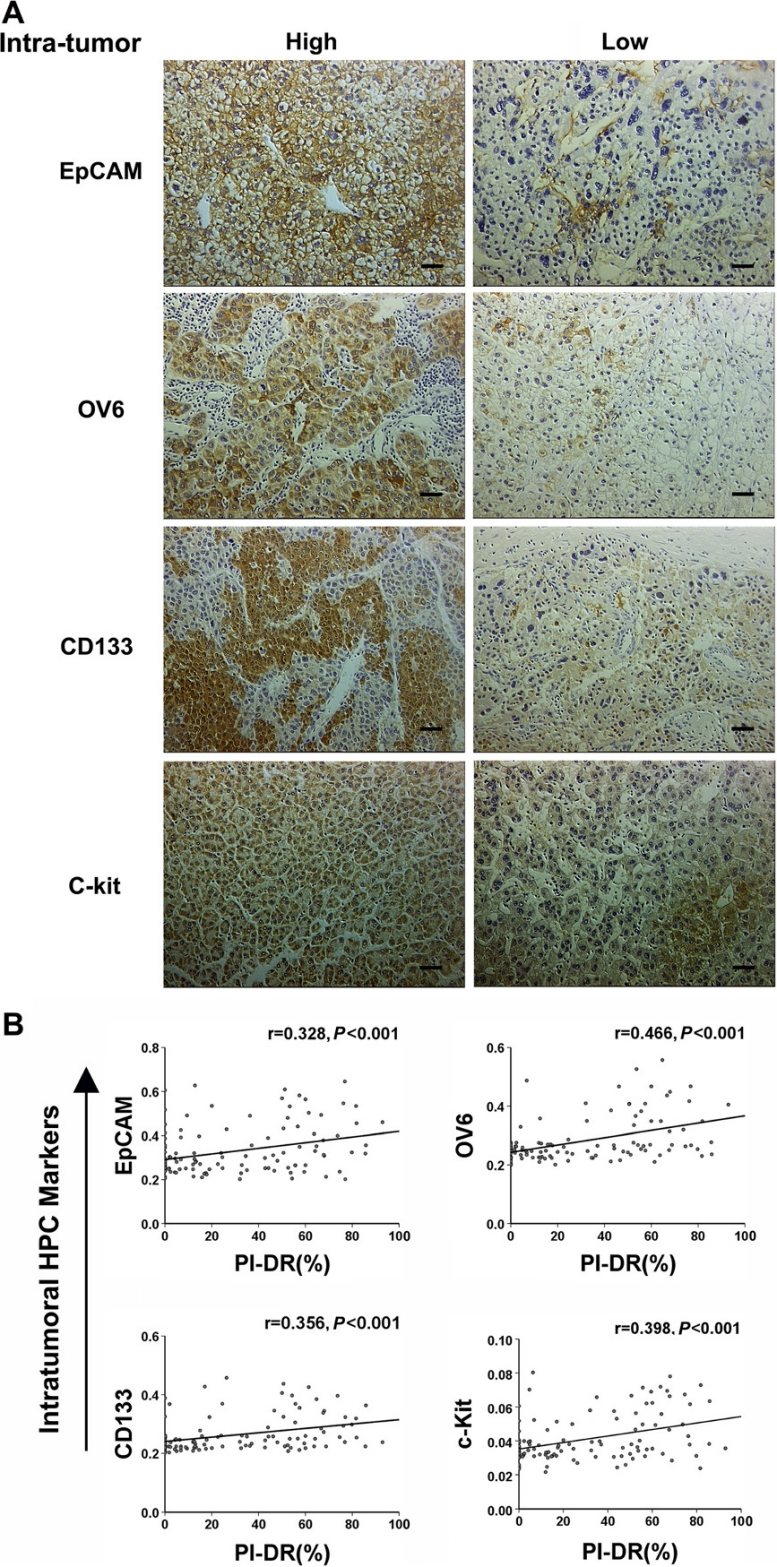
**The peritumoral PI** **-DR positively correlates with the intratumoral HPC markers expression.** **(A)** Photographs of immunostaining of hepatic progenitor cell markers in intratumoral sections of tissue microarrays were taken for analyses (sacle bar =100 μm) . High and low density of EpCAM, OV6, CD133 and c-kit expression in intratumoral sections. **(B)** Scatterplot with fitting line shows peritumoral PI-DR positively correlates with the intratumoral EpCAM, OV-6, CD133 and c-kit expression measured by IOD. Pearson correlation analysis provides correlation coefficient (r) and *P*-value (right).

To assess if the intratumoral expression of HPC markers were correlated with PI-DR in our study, we further performed an IHC analysis of four HPC markers (EpCAM, OV6, CD133 and c-kit) in the intratumoral tissue sections of the entire group as shown in Figure 3A, the high intratumoral expression of EpCAM, OV6, CD133 and c-kit was seen in 39 of 120 (32.5%), 26 of 120 (21.7%), 29 of 120 (24.2%), and 34 of 120 (28.3%) cases, respectively (Table 3).

**Table 3:** **Immunohistochemical stain results and clinicopathologic features in intratumoral tissue of 120 HCCs**

Clinicopathologic features	Intratumoral EpCAM density		***P***Value	Intratumoral OV6 density		***P***Value	Intratumoral CD133 density		***P***Value	Intratumoral c-kit density		P Value
	High	Low		High	Low		High	Low		High	Low	
Frequency (%)	39 (32.5%)	81 (67.5%)		26 (21.7%)	94 (78.3%)		29 (24.2%)	91 (75.8%)		34 (28.3%)	86 (71.7%)	
Gender												
Male	34	74	0.697	21	87	0.161	26	82	1.000	32	76	0.543
Female	5	7		5	7		3	9		2	10	
Age												
≤60	19	44	0.565	19	62	0.493	13	50	0.342	19	44	0.641
>60	20	37		7	32		16	41		15	42	
AFP												
≤400	17	64	<0.001*	19	62	0.493	20	61	0.847	23	58	0.983
>400	22	17		7	32		9	30		11	28	
Cirrhosis												
Absence	21	50	0.411	10	61	0.015*	14	57	0.171	17	54	0.199
Presence	18	31		16	33		15	34		17	32	
Satellite lesion												
Absence	33	72	0.507	22	83	0.867	27	78	0.468	33	72	0.092
Presence	6	9		4	11		2	13		1	14	
Tumor size (cm)												
≤5cm	15	37	0.455	14	38	0.222	13	39	0.852	12	40	0.264
>5cm	24	44		12	56		16	52		22	46	
Tumor encapsulation												
Absence	26	61	0.321	18	69	0.673	19	68	0.334	27	60	0.286
Presence	13	20		8	25		10	23		7	26	
Microvascular invasion												
Absence	15	40	0.261	14	41	0.354	12	43	0.580	17	38	0.565
Presence	24	41		12	53		17	48		17	48	
Major vascular invasion												
Absence	34	80	0.023*	25	89	1.000	28	86	1.000	32	82	1.000
Presence	5	1		1	5		1	5		2	4	
Edmondson grade												
Low (I/II)	23	67	0.005*	17	73	0.201	19	71	0.176	20	70	0.010*
High (III/IV)	16	14		9	21		10	20		14	16	
TNM stage												
I/II	27	65	0.181	17	75	0.124	20	72	0.260	28	64	0.354
III	12	16		9	19		9	19		6	22	

*p <0 .05 was considered statistically significant.

HCCs were grouped according to intratumoral HPC expression, and clinicopathological features were compared as shown in the Table 3. High intratumoral EpCAM was significantly associated with more major vascular invasion (*P* = 0.023), high AFP (*P* < 0.001) and high Edmondson grade (*P* = 0.005), High intratumoral OV6 expression of the entire group demonstrated more cirrhosis (*P* = 0.015). High intratumoral c-kit expression was significantly more likely to occur in high Edmondson grade (*P* = 0.010).

We further analyzed the correlation of PI-DR with the intratumoral HPC markers’ expression (IOD). PI-DR had a positive correlation with intratumoral EpCAM (r = 0.328, *P* < 0.001, OV6 (r = 0.466, *P* < 0.001), CD133 (r = 0.356, *P* < 0.001) and c-kit expression (r = 0.398, *P* < 0.001) (Figure 3B) in the entire group. Thus, together these results suggest that PI-DR could be used as a valid surrogate for intratumoral expression of HPC markers.

### The association of peritumoral high PI-DR in HCC with poor disease-free survival

**Figure 4:**
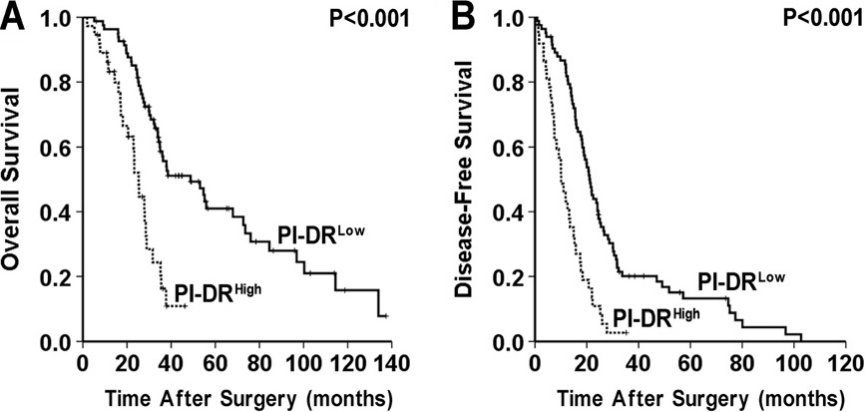
**Cumulative overall and disease**-**free survival curves of HCC patients with high or low PI**-**DR.** **(A)** Low PI-DR in peritumoral tissue was associated with prolonged overall survival. **(B)** High PI-DR in peritumoral tissue was associated with poor disease-free survival. Low PI-DR: (<50%); High PI-DR (≥50%).

The survival of the selected HCC patients was analyzed with Kaplan-Meier survival analysis. Patients with high PI-DR (≥50%) were likely to be with significantly poor OS and DFS. The median (95% CI) overall survival time was 25.3 (21.7-28.8) and 48.8 (31.3-66.3) months respectively for patients with high PI-DR (≥50%) and low PI-DR (<50%) in HCC (*P* < 0.001, Figure 4A). The median (95% CI) disease-free survival time was 10.1 (7.0 - 13.2) and 21.2 (18.4 - 24.1) months respectively for patients with high PI-DR (≥50%) and low PI-DR (<50%) in HCC (*P* < 0.001, Figure 4B).

### Univariate and multivariate analyses of prognostic variables in HCC patients

In univariate analysis, PI-DR (≥50% vs < 50%), K7-DR (≥50% vs < 50%), TNM stage (I/II vs. III), macrovascular invasion (presence vs. absence) and intratumoral EpCAM Expression (High vs. Low) showed a significant association with poor overall survival (*P* < 0.001, *P* = 0.045, *P* = 0.030, *P* = 0.014 and *P* = 0.017 respectively, Table 4). Univariable analysis also revealed that PI-DR (*P* < 0.001), TNM stage (I/II vs. III) (*P* = 0.001), Edmondson grade (I/II vs. III/IV) (*P* = 0.043), peritumoral EpCAM expression (High vs. Low) (*P* = 0.003), peritumoral CD133 expression (High vs. Low) (*P* < 0.001), intratumoral OV6 expression (High vs. Low) (*P* = 0.034) were independent prognostic factors for DFS after resection (Table 4).

**Table 4:** **Univariate and multivariate analyses of factors associated with survival and recurrence in hepatocellular carcinoma group of testing cohort** (**n** = **120**)

Factors			Overall Survival (OS)		Disease-free Survival (DFS)		
		Univariate	Multivare		Univariate	Multivariate	
		***P***	HR (95% CI)	***P***	***P***	HR (95% CI)	***P***
Characteristic of	Age: >50 vs. ≤50 years*	0.719		NA	0.981		NA
Demography,	Gender: male vs. female	0.846		NA	0.088		NA
Tumor &	Tumor size: >5 vs. ≤5 cm	0.061		NA	0.130		NA
Laboratory	Tumor number: multiple vs. single	0.221		NA	0.192		NA
Tests	Serum AFP: ≥400 vs. <400 ng/ml	0.285		NA	0.382		NA
	Serum CA199 ≥ 37 vs. < 37 U/ml	0.798		NA	0.236		NA
	TNM stage: I/II vs. III	0.030*		NS	0.001*	1.817 (1.135-2.909)	0.013*
	Edmondson grade: I/II vs. III/IV	0.074		NA	0.043*		NS
	Macrovascular Invasion: P vs. N	0.014*		NS	0.114		NA
	Tumor encapsulation: absence vs. presence	0.791		NA	0.311		NA
Histology:	Ishak grade >7 vs. ≤ 7	0.058		NA	0.072		NA
Peritumor	Ishak stage >5 vs. ≤ 5	0.096		NA	0.120		NA
	PI-DR ≥ 50% vs. <50%	<0.001*	3.316(1.961-5.610)	<0.001*	<0.001*	2.618(1.701-4.029)	<0.001*
	K7-DR ≥ 50% vs. <50%	0.045*	1.863(1.008-3.443)	0.047*	0.158		NA
	EpCAM^Peritumor^ Expression H vs. L*	0.319		NA	0.003*		NS
	OV6^Peritumor^ Expression H vs. L*	0.191		NA	0.702		NA
	C-kit^Peritumor^Expression H vs. L*	0.721		NA	0.935		NA
	CD133^Peritumor^ Expression H vs. L*	0.051		NA	<0.001*		NS
Histology:	Microvascular invasion: P vs. N	0.327		NA	0.595		NA
Tumor:	EpCAM Expression H vs. L*	0.017*		NS	0.093		NA
	OV6 Expression H vs. L*	0.756		NA	0.034*		NS
	C-kit Expression H vs. L*	0.352		NA	0.127		NA
	CD133 Expression H vs. L*	0.394		NA	0.091		NA

*P*, positive; *N*, negative; *H*, High; *L*, Low; *NA*, not adopted; *NS*, not significant;*AFP*, alpha-fetoprotein; *ALT*, alanine aminotransferase; *CA*19-9, carbohydrate antigen 19–9; *CI*, confidence interval; *PI*-*DR*, proliferative index of ductular reaction; *DR*, ductular reaction; EpCAM, epithelial cell adhesion molecule; *HCC*, hepatocellular carcinoma; *HR*, hazard ratio; K7-*DR*, Keratin 7 immunoreactive ductular reaction; *TNM*, tumor-node-metastasis;

*Medians were used for cutoff values.

Factors showing significance by univariate analysis were adopted when multivariate Cox proportional hazards analysis was performed (Table 4). PI-DR (HR: 3.316, 95% CI: 1.961 - 5.610, *P* < 0.001), K7-DR (HR: 1.863, 95% CI: 1.008-3.443, *P* = 0.047) were independent prognostic factors for overall survival. Moreover, PI-DR (HR: 2.618, 95% CI: 1.701 - 4.029, *P* < 0.001) and TNM stage (HR: 1.817, 95% CI: 1.135 - 2.909, *P* = 0.013) were also independent predictors for disease-free survival.

## Discussion

The main findings of our study are that in a cohort of patients with HCC (1) the peritumoral proliferative index of DR (PI-DR) stained with PCNA is observed within the HCCs and the PI-DR correlates closely with the necroinflammation grade and fribrosis stage. (2) The PI-DR correlates strongly with the expression of peritumoral and intratumoral HPC markers in HCC patients. (3) PI-DR is the independent risk factor for both DFS and OS in HCCs.

It is well known that the important consequences of the ductular reaction are inflammatory infiltration, rich in neutrophil inflitration (cholangiolitis), periportal fibrosis and neovascularization [20]. The extensive analysis supported that the DRs correlated closely with severity of fibrosis across a range of liver pathologies including chronic hepatitis C, alcoholic and non-alcoholic steatohepatitis, and fibrosing cholestatic hepatitis [12]. In our study, that is also evidenced by high levels of proliferation rate within DRs strongly correlating with necroinflammation and fibrosis in HCC patients. Our study does not prove that the proliferative ductular reaction directly promote necroinflammation and fibrosis, or that the reaction is simply a by-product of necroinflammation and fibrosis. Clouston [9] and colleagues found that the presence of a fine ductular reaction in some patients without portal fibrosis, may suggest that the ductular reaction itself may be important in the development of fibrosis.

In normal liver undergoing partial hepatectomy or acute injury, hepatocyte-mediated regeneration predominates. In chronic and severe injury, however, ductular reactions (DRs) of activated biliary epithelial cells which contain hepatic progenitor cells (HPCs) appear in the periportal regions. In our study, we studied the proliferative state of ductular reaction, with PCNA as a proliferation marker, to test the hypothesis that whether the proliferative ductular reaction (PI-DR) correlated with the expression of peritumoral and intratumoral HPC markers. We identified PI-DR had positive correlations with EpCAM, OV6, CD133 and c-kit expression in both peritumoral and intratumoral tissue of the entire group. Our data indicated that proliferative ductular reaction could serve as a valid surrogate for peritumoral and intratumoral expression of HPC markers. Consistent with our findings, Cai et al. [21] have found that proliferative ductular reaction is related to HPC activation in patients with combined hepatocellular-cholangiocarcinoma. Lennerz and colleagues [22] suggested marked alterations in cellular identity as an underlying mechanism for the reproducible perinodular DR that parallels progressive stages of intranodular hepatocarcinogenesis. Several studies have also shown that high levels of proliferation rate within DRs has been demonstrated in both oval cell-mediated liver regeneration in an animal model and human liver disease [23, 24]. The other published studies have shown that ductular reaction is frequently associated with fibrosis and inflammation in chronic liver disease and may be key factors promoting liver progenitor cell expansion in alcoholic hepatitis [25]. Delladetsima et al. [26] have reported a periportal ductular reaction was noted in association with HPC expansion in HCV.

In the present study, we also conducted an immunohistochemical analysis of four HPC markers (EpCAM, OV6, CD133 and c-kit) in peritumoral tissue microarray, and compared the various clinicopathologic features according to the expression status of each of these markers. In our study, univariate survival analysis demonstated that peritumoral EpCAM expression and peritumoral CD133 expression were independent predictors of disease-free survival. This stands in consistent with findings reported by Hoshida et al. [27], their group disclosed that gene expression profiles of HCC failed to yield a significant association with survival, whereas surrounding nontumoral profiles correlated with recurrence. Moreover, in our data the intratumoral expression of HPC markers’ expression were also studied in the microarray tissue of the HCCs. Univariate survival analysis also demonstated that intratumoral EpCAM expression was an independent predictor of overall survival. Bae et al. [28] have also demonstrated EpCAM expression occurs at distinct nodular stage of HCC and could play an important role in HCC progression.

High incidence of intrahepatic metastasis and recurrence after resection suggested that peritumoral environment is an important but often neglected issue. In our previous study, Xu et al. [8] have uncovered peritumoral DR in a necroinflammatory microenvironment correlates with postoperative prognosis in HCC. Richardson et al. [10] found that an altered replication pathway in active nonalcoholic steatohepatitis promotes a periportal DR, which in turn may provoke progressive periportal fibrogenesis. The main finding of our study is that the peritumoral proliferative state of DR (PI-DR) is correlated with necroinflammation grade and fribrosis stage, and PI-DR may be involved in the HPC-derived carcinogenesis in the progression of HCC. Our finding are in accord with the following prior published data regarding the proliferative state of ductular reaction. Cai et al. [21] in our lab also identified that a “progenitor dominant” regeneration pattern, which is closely related with intensive transit-amplifying DR, is associated with the poorest OS and DFS. Yoon et al. [29] found the cells of ductular reactions showed markedly elevated proliferation rates in patients with worsening stage of chronic hepatitis B and C. Importantly in our study, in Cox regression analysis including all clinico-pathological covariates for analysis, peritumoral PI-DR was the independent predictor for overall and disease-free survival in HCC.

Taken together, our results demonstrated that peritumoral PI-DR, strongly correlated with both the peritumoral and intratumoral HPC expression, may be associated with subsequent tumor development. PI-DR could be a useful marker for prognostic prediction of recurrence in HCC.

## Conclusion

In summary, we found that in HCC patients, the peritumoral DR with higher PCNA labeling index was prone to correlate with the degree of necroinflammatory activity and the stage of fibrosis. We also demonstrated the peritumoral PI-DR correlated stongly with both the peritumoral and intratumoral expression of HPC markers, and peritumoral PI-DR predicted reccurrence in HCC after hepatectomy. These findings suggested that the PI-DR which strongly correlated with HPC markers could represent an early stage of carcinogenesis in HCC , that would be targeted and could help define HCC patients at high risk for developing HCC after hepatectomy. The potential mediators of PI-DR are of great interest and warrant further study.

## Materials and methods

### Patients and clinical data

Archived, formalin-fixed and paraffin-embedded liver specimens used in this study were obtained from the Eastern Hepatobiliary Surgery Hospital, Shanghai, China. From January 1997 to December 2007, patients who underwent hepatectomy and who were post-operatively confirmed as hepatocellular carcinoma were recruited for prospective follow-up. Informed consent was obtained from each patient under a protocol approved by the Hospital Research Ethics Committees. The following tissue microarray included 138 pairs of intratumoral and peritumoral liver tissues from the selected HCC patients. Eighteen cases could not be analysed because of the loss of the samples during the preparation of the tissue microarray. The 120 remain study population consisted of 108 men and 12 women with a mean age of 50.1 ± 11.9 years (range 11–89 years). Tumor size was based on the largest dimension of the tumor specimen. Satellite lesions were defined by the presence of 2 or more nodules including intrahepatic metastases. Microvascular invasion was determined by microscopic examination of the resected specimen, whereas macrovascular invasion was identified by macroscopic examination of specimen. Tumor stage was determined according to the 2009 UICC TNM (International Union Against Cancer, Tumor Node Metastasis) classification system [30]. Tumor differentiation was assessed according to Edmonson and Steiner grading system. The severity of the inflammation at the interface between portal tracts and the parenchyma was assessed on the H&E stained section and graded according to Knodell et al. [31]. Fibrosis was assessed separately, as suggested by Desmet et al. [32]. The specimens were divided into four groups according to the HAI score for necroinflammation grade. The first group included biopsies with HAI 0–4 (absent activity), the second group biopsies with HAI 5–8 (mild activity), the third group biopsies with HAI 9–12 (moderate activity) and the last group biopsies with HAI 13–18 (severe activity). The specimens were also divided into three groups according to the degree of fibrosis, which was staged with scores ranging from 1 to 3, corresponding to: absence or mild fibrosis (stage 1), moderate or severe (bridging) fibrosis (stage 2) and cirrhosis (stage 3).

### Follow-up and detection of recurrence

All patients were followed regularly every 2–3 months after surgery until study closure in July 2010 with serum AFP/CA19-9 and abdominal ultrasonography. Progressive elevation of serum AFP/CA19-9 levels and/or ultrasonographic detection of a new hepatic lesion prompted hospitalization for confirmation of diagnosis and appropriate management, including repeat resection, radiofrequency ablation (RFA), transcatheter arterial chemoembolization (TACE) or supportive therapy. Recurrence was confirmed by contrast-enhanced imaging studies according to standard guidelines for HCC [33]. Overall survival (OS) was defined as the interval between the dates of surgery and death while disease-free survival (DFS) was defined as the interval between the dates of surgery and recurrence. If recurrence was not diagnosed, patients were censored on the date of death or the last follow-up. Clinical follow-up was not disclosed to laboratory personnel until statistical analysis.

### Tissue microarray (TMA) construction

Tissue microarray (TMA) construction was described in previous reports [34]. Briefly, we constructed the tissue microarray, containing of 120 pairs of intratumoral and peritumoral liver specimens from the enrolled HCC patients. TMA slides with 120 pairs of intratumoral and matched peritumoral samples were constructed. In brief, all archival formalin-fixed and paraffin-embedded specimens used in this study were freshly sectioned and stained with haematoxylin and eosin (H & E). The H & E-stained sections were carefully reviewed by two independent pathologists without knowledge of the patients’ clinical characteristics and outcomes, and the representative regions of the intratumoral and peritumoral liver tissues were defined for microarray, and the peritumoral tissues at the distance (15mm) from the tumor margin were collected. Using a tissue arraying instrument (Beecher Instruments, Sliver Spring, MD), each tissue core with a diameter of 1.5 mm was punched from the marked areas of intratumoral and peritumoral tissues and re-embedded. TMAs containing the tissue cores were then cut into five-micrometre sections for IHC staining.

### Immunohistochemical staining

Serial five-micrometre thick sections of tissue array blocks were examined immunohistochemically using a standard two-step method. Sections were de-paraffinized and rehydrated, and endogenous peroxidase was quenched with 3% peroxid (20 min). After antigen retrieval in 10mM sodium citrate buffer (pH 6.0) or Ethylene Diamine Tetraacetic Acid (EDTA,1 mmol/L, PH 8.0) for 20 min at 95°C, the sections then were treated with 2% normal goat serum and incubated with primary antibodies overnight at 4°C. After washing with phosphatebuffered saline, sections were incubated with Two stept anti-rabbit or anti-mouse reagent (HRP) (Antibody, Diagnostica Inc, NY) at 37°C for an hour. 3,3′-Diaminobenzidine tetrahydrochloride (DAB) was used as the suitable substrate-chromogen and Mayer’s hematoxylin counterstain was applied. Negative controls were treated identically but with the primary antibody omitted. Information on antibodies used and antigen-retrieval conditions are summarized in Table 5.

**Table 5:** **List of antibodies used**

Antibody	Source	Dilution	Antigen retrieval
CK7	Mouse monoclonal; Dako, Glostrup, Denmark	1:50	Citrate buffer, pH 6.0
EpCAM	Mouse monoclonal; Dako, Glostrup, Denmark	1:200	Citrate buffer, pH 6.0
CD133	Rabbit monoclonal; Cell Signaling Technology, Danvers, MA	1:100	Citrate buffer, pH 6.0
CD117 (c-kit)	Rabbit polyclonal; Dako, Glostrup, Denmark	1:400	EDTA buffer, PH 8.0
OV6	Mouse monoclonal; R & D Systems, Emeryville, CA	1:40	EDTA buffer, PH 8.0
PCNA	Mouse monoclonal; Cell Signaling Technology, Danvers, MA	1:4000	Citrate buffer, pH 6.0

*CK*7 cytokeratin 7, *EpCAM* epithelial cell adhesion molecule , *CD*133 cluster of differentiation (CD)133, CD117 (c-kit) mast/stem cell growth factor receptor, *OV*6 oval cell marker, *PCNA* proliferating cell nuclear antigen.

### Histopathologic and immunohistochemical evaluation

All slides of positive HPC markers (EpCAM, OV6, C-kit and CD133) staining were observed and photographed with an Olympus microscope (IX-70 OLYMPUS, Japan). Three independent investigators examined the TMA slides without related clinical information. Under 200× magnification, images of positive representative fields were captured. The density of immunostaining was measured using Image-Pro Plus Version 6.2 software (Media Cybernetics Inc., Bethesda, MD), described previously as an established method [4]. The integrated optical density (IOD) in each image was measured, and the density of HPC markers was calculated as IOD/total area of each image. For calculating HPC markers (e.g. EpCAM, OV6, CD133 and c-kit) density, the cutoff for the definition of subgroups was the median IOD to separate high HPC markers’ expression (IOD > median) from low HPC markers’ expression (IOD ≤ median) in microarray tissue samples.

### Evaluation of K7-DR and PI-DR

For quantification of the total area of ductular reaction (DR), non-overlapping fields of the biopsy specimen were photographed at 100× magnification after immunohistochemical staining for CK7. The extent of CK7-immunoreactive ductular reaction was semiquantitatively assessed and graded with some modification in a manner analogous to the grading of interface hepatitis in the scoring system of Ishak et al. [31]: In addition, proliferation rate in DR was evaluated by calculating the proliferating cell nuclear antigen(PCNA) labeling index as described in our previous reports [21]. Specifically, 3 400× highpowered fields within each section were randomly chosen and captured. The same fields were captured in sequential serial sections stained with K7 for quantification of the number of reactive ductular cells (RDCs) in reactive ductules [35]. The proliferation index of DR (PI-DR) was calculated as ratio between the number of PCNA immunoreactive nuclei and the total number of RDCs.

### Statistical analysis

Statistical analysis was performed using with SPSS 20.0 for Windows (SPSS Inc., Chicago, IL); Differences between categorical variables were assessed by the chi-square test or Fisher’s exact test, when necessary. Pearson’s correlation coefficient was used to determine correlations between continuous normally distributed variables. The degree of association between nonparametric or ordinal variables was assessed by using Spearman nonparametric correlation. Kaplan-Meier analysis was used to determine the survival. Log-rank test was used to compare patients’ survival between subgroups; Significant variables from the univariable analysis were entered in the multivariable analysis, which was performed using the Cox-proportional hazards model with forward stepwise selection. P < 0.05 was considered statistically significant.

## Abbreviations

HCC: Hepatocellular carcinoma
HPCs: Hepatic progenitor cells
DR: Ductular reaction
PI-DR: Proliferative index of ductular reaction
EpCAM: Epithelial cell adhesion molecule
CD133: Cluster of differentiation (CD)133
DFS: Disease-free survival
OS: Overall survival
PCNA: Proliferating cell nuclear antigen.
